# Supplementary material for: Labour admission assessment results of index pregnancy as predictors of intrapartum stillbirth in public health facilities of Addis Ababa: A case-control study
Source: PLoS One. 2020 Apr 2;15(4):e0230478. doi: 10.1371/journal.pone.0230478 (PMC7117700; doi:10.1371/journal.pone.0230478)
Supplement: S1 Data — (DOCX) [file pone.0230478.s001.docx]

Labour admission assessment results of index pregnancy as predictors of intrapartum stillbirth in public health facilities of Addis Ababa: a case-control study

Authors

1. Alemayehu Gebremariam Agena, PhD

Department of Health Studies, University of South Africa

Tel - +250788309289; e-mail – [alemayehug2000@gmail.com](mailto:alemayehug2000@gmail.com)

P.O.Box 65, Kigali, Rwanda

Corresponding author

1. Prof. Lebitsi M. Modiba, Professor

Department of Health Studies, University of South Africa, TvW 7-160 College of Human Sciences, Tel: 012 429 6337; E-mail: [modiblm@unisa.ac.za](mailto:modiblm@unisa.ac.za)

Original Manuscript _Reproductive Health

Labour admission assessment results of index pregnancy as predictors of intrapartum stillbirth in public health facilities of Addis Ababa: a case-control study

Alemayehu Gebremariam Agena^1*^; Lebitsi M. Modiba^2^

### **Abstract**

**Background**

Approximately one-third of the global stillbirth burden occurs during intrapartum period. The ability to assess obstetric parameters including effacement, dilatation, uterine contraction, decent, rupture of the uterus, and moulding of the foetal head are among the essential competencies required by obstetric service providers admitting women for labour in health facilities. Misdiagnosis of these conditions could result in unnecessary obstetric interventions and unfavourable obstetric outcomes including intrapartum stillbirth. This study aimed to assess associations between accurate diagnosis of labour on admission and intrapartum stillbirth.

**Methods**

A case-control study using primary data from chart review of medical records of women who experienced intrapartum stillbirth in 20 public health centres and three public hospitals of Addis Ababa between 01 July 2010 and 30 June 2015 was conducted. Data were collected from charts of all cases meeting the inclusion criteria. Medical records of women with livebirths were randomly selected and reviewed from each public health facilities in two to one (2:1) control to case ratio. Accordingly, 728 cases of stillbirth out of 1,056 charts met the inclusion criteria whereas 1,551 controls out of 1,705 were also considered in the study.

**Results**

Proportionally, more women in the stillbirth group (39.4%) than in the livebirth group (30.2%) experienced ruptured membrane on admission, with the difference being statistically significant (OR 1.7, 95% CI 1.37–2.03). Significantly higher proportion of women in the intrapartum stillbirth group experienced FRH lower than 110/min, a result suggestive of foetal distress on admission. Proportionally, more women in the intrapartum stillbirth group (14.5%) than in the livebirth group (4.5%) had breech foetal presentation on admission for labour, the difference being statistically significant (aOR 3.26 95% CI 1.93-5.50). Intrapartum stillbirth was significantly higher among women with cervical dilatation 4cm or more on admission (OR 1.2, 95% CI 1.00–1.45). This could be owing to delay in seeking obstetric care or misdiagnosis of the condition, a situation that seeks more rigorous study to determine the underlying causal links.

**Conclusion**

Low FHR, non-vertex foetal presentations and ruptured cervical membrane were predictors of intrapartum stillbirth. Health facilities could avert unnecessary foetal loss by undertaking timely and correct diagnosis of admission-related obstetric indicators.

^*^ Corresponding author – alemayehug2000@gmail.com

**Plain English Summary**

This study was conducted in the public health facilities of Addis Ababa to assess if the clinical observations related to indicators of labour commencement for women admitted for childbirth could predict the occurrence of stillbirth during childbirth. Medical records of all women who experienced stillbirth and equal number of women who had normal birth were reviewed on four indicators. These include rupture of the membrane that encapsulated unborn babies, the rate of heartbeat of unborn babies, the dilatation of the cervix, and the presenting part of the baby that descends through the birth canal. The assessment covered 20 public health centres and three public hospitals and medical records were reviewed retrospectively for five-year period from July 2010 – June 2015.

The results showed that women who experienced stillbirth during childbirth had clinically poor performance on the labour indicators at admission compared to women in the normal birth group. For instance, the heartrate of babies who died during the childbirth was substandard, proportionally more women in the stillbirth groups had raptured membrane, and more babies in the stillbirth group descended with abnormal presentations during admission to labour.

In general, substandard foetal heartrate, abnormal foetal presentations and ruptured cervical membrane were predictors of intrapartum stillbirth. Health facilities could avert unnecessary foetal loss by undertaking timely and correct diagnosis and by applying emergency childbirth skills promptly.

# **Background**

Approximately one million intrapartum stillbirths occur annually, representing one-third of the total stillbirth burden globally (1). Despite the caveats inherent in the interpretation of the intrapartum stillbirth estimates, the situation clearly shows the magnitude of tragic loss of life just minutes and hours prior to birth (2). Evidence shows that most of these stillbirths can be prevented through the correct application of clinical and obstetric skills; hence, the current high prevalence is unacceptable (3).

A human pregnancy lasts approximately 40 weeks with the anticipation of a normal labour occurring between 37–40 weeks of gestations. Normal labour is considered to be of relatively low risk and usually starts spontaneously with vertex presentation of the foetus and culminates in the ejection of a live baby whereas the woman stays in healthy condition (4). The onset of labour is determined by complex physiological interactions and its diagnosis requires important clinical and midwifery competence. The ability to correctly diagnose clinical parameters including the effacement and dilatation of cervix, strength of uterine contraction, decent of the head of the foetus, rupture of the uterus, and status of amniotic fluid and moulding of the foetal head are among the essential competencies required during admission to labour.

Many clinicians view 3 or 4 cm cervical dilation as the beginning of active phase of labour including the WHO’s partograph which takes 4cm as the benchmark for the commencement of active phase of labour. However, the American College of Obstetricians and Gynaecologists (ACOG) postulates that labour progresses at a rate substantially slower than historically believed and that a cervical dilatation of 6 cm should be considered as a threshold for the active phase of most women in labour (5).

Skilled birth attendants are expected to make comprehensive assessment of pregnant women on arrival for labour to decide on the required intensity of follow-up and obstetric interventions. Making accurate diagnosis of the maternal and foetal conditions on admission for labour based on the above indicators depends on the competency of the obstetric care providers, maternal characteristics and availability of necessary supplies and equipment at the health facilities.

On the contrary, the misdiagnosis of these conditions could result in unfavourable outcomes and unnecessary obstetric interventions. For instance, early admission to labour was associated with a significantly higher risk of delivery by caesarean section during the first and second stages (6). Furthermore, evidence shows that the proportion of pregnant women who received key interventions including augmentation with oxytocin, artificial rupture of membranes and caesarean section were significantly higher in the latent phase group than in the active phase group which shows misdiagnosis of labour progress can result in untimely interventions. Spontaneous vertex delivery was significantly higher in the active phase group than the latent phase group (7).

This study collected data from the public health facility in Addis Ababa on key obstetric conditions including the status of membrane, FHR, cervical dilatation, and foetal presentation on admission to labour to assess if these indicators were correctly observed and had any associations with intrapartum stillbirth compared to livebirth.

**Method and Materials**

**Study setting and design**

This was a case-control study using primary data from chart review of medical records of women who experienced intrapartum stillbirth in 20 public health centres and three public hospitals of Addis Ababa during the period 01 July 2010 – 30 June 2015. In 2010, 26 public health centres offered Basic Emergency Obstetric and Neonatal Care (BEmONC) in Addis Ababa (8) out of which 20 were selected for this study based on case volume. Similarly, chart reviews were conducted in three out of the five public hospitals under the Addis Ababa City Administration, where Comprehensive Emergency Obstetric and Neonatal Care (CEmONC) had been practiced since 2010.

**Study Population and Sampling**

All cases of intrapartum stillbirths that occurred in the 20 public health centres and three public hospitals in Addis Ababa and recorded in the maternity registers of respective facilities were considered for this study. Given intrapartum stillbirth is a relatively rare phenomenon, this study included all cases of intrapartum stillbirths meeting the inclusion criteria. The most important inclusion criterion was the fact the foetus was alive up on admission to labour in the respective health facilities, which constitutes the basis for definition of intrapartum stillbirth.

Controls were selected randomly from the same maternity registers which constituted a sampling frame in each public health facilities using a lottery method until two to one (2:1) control to case ratio was achieved. On every page where cases of intrapartum stillbirth were detected, record numbers of women with livebirth were listed and rolled on pieces of paper of which an individual other than the data collector randomly selected charts until the required number of controls achieved.

**Data collection and Analysis**

Quantitative data on key variables related to labour admission assessment results were collected from the admission records of women who had given birth in the public health facilities in Addis Ababa from 01 Jul 2010 – 30 June 2015. Data entry and analysis were conducted using SPSS version 24 from 01 August – 30 Sept 2016. Bivariate analysis was conducted for key independent variables followed by multivariate logistic regression model for variables with p-value of 0.02 and less.

**Ethical Considerations**

Data were collected from medical records thereby minimising the concerns of confidentiality and requirements for individual consents. The data collector was trained and strictly supervised on adhering to the principles of confidentiality of clients' information on the records during the process of data collection. The chart review was conducted within the respective facilities through consented authorisation of relevant facility leadership. Individual data sources remained anonymous during analysis and report writing. Furthermore, ethical approval was obtained from the Higher Degrees of the University of South Africa (HSHDC/421/2015), and study permit was secured from Health Ethics Committee of Addis Ababa Regional Health Bureau (AARHB) prior to data collection.

**Results**

Of the documented 112 intrapartum stillbirth cases in the 20 public health centres in Addis Ababa, 91 (81%) met the selection criteria and were included in this study. Similarly, there were a total of 944 cases of intrapartum stillbirth in the three public hospitals of which 637 (67%) qualified the inclusion criteria. A total of 427 chart of controls were reviewed in the 20 public health centres of which only 273 (64%) were included. Moreover, 1738 controls were also randomly identified in the three public hospitals of which 1278 (74%) qualified the inclusion criteria. In general, 728 cases of intrapartum stillbirth and 1551 controls were considered from all the target public health facilities in Addis Ababa.

**Socio-demographic characteristics**

Data were collected on five key socio-demographic variables including age, marital status, gravida, para, and number of children alive for the women whose charts were reviewed in this study. Accordingly, approximately 57% of women who experienced intrapartum stillbirth and 60% who had livebirths reported to be in the age category 25–34 years. The second highest proportion of women in the study population for both intrapartum stillbirth (35.8%) and livebirth (33.6%) was found in the age group 15–24 years. Results from this study showed that proportionally more women in the stillbirth category (49.3%) than in the livebirth (37.1%) conceived for the first time. Consistent with the results on gravida, intrapartum stillbirth was proportionally more common among primigravida (60%) compared to those who gave birth to up to three children. This study did not reveal any statistically significant differences between stillbirth and livebirth categories for women of three and higher birth orders.

Table 1: Distribution of Socio-Demographic Characteristics among Cases and Controls

| **Characteristic** | **Stillbirth**  **N (%)** | **Livebirth**  **N (%)** |  |
| --- | --- | --- | --- |
| **Age (Years)** |  |  |  |
| 15-24 | 261(35.8) | 522(33.6) |  |
| 25-34 | 416(57.2) | 931(60.3) |  |
| 35-49 | 51(7.0) | 98(6.1) |  |
| **Marital Status** |  |  |  |
| Married | 314(42.7) | 982(64.4) |  |
| Divorced | 3(0.4) | 5(0.3) |  |
| Widowed | 0(0.0) | 3(0.2) |  |
| Separated | 0(0.0) | 2(0.1) |  |
| Never Married | 11(1.5) | 43(2.8) |  |
| Missing | 400(54.9) | 516(33.2) |  |
| **Gravida** |  |  |  |
| One | 360(49.3) | 575(37.1) |  |
| Two | 203(28.0) | 539(34.8) |  |
| Three | 84(11.5) | 256(16.5) |  |
| Four | 55(7.6) | 133(8.6) |  |
| Five and Above | 26(3.7) | 48(3.0) |  |
| **Para** |  |  |  |
| Zero | 442(60.3) | 744(48.1) |  |
| One | 185(25.4) | 542(35.0) |  |
| Two | 57(7.9) | 177(11.4) |  |
| Three | 31(4.3) | 61(3.9) |  |
| Four | 10(1.5) | 19(1.2) |  |
| Five and Above | 4(0.5) | 8(0.5) |  |
| **Children** |  |  |  |
| Zero | 451(68.8) | 790(55.2) |  |
| One | 134(20.4) | 435(30.4) |  |
| Two | 43(6.6) | 139(9.7) |  |
| Three | 21(3.2) | 49(3.4) |  |
| Four and Above | 7(1.1) | 17(1.2) |  |
| Missing | 72(9.8) | 121(7.8) |  |
|  |  |  |  |

### **Labour admission assessment results**

This study collected data on four indicators, namely, status of membrane, FHR, cervical dilatation, and foetal presentation on admission to examine their association with intrapartum stillbirth in the targeted public health facilities in Addis Ababa. Accordingly, 610 (83.8%) cases of stillbirth and 1,347 (86.8%) livebirth women were assessed for the status of membrane on admission. Out of these, 46.8% of intrapartum stillbirth and 59.9% of livebirth women had intact membranes on admission for labour.

On the contrary, proportionally more women in the intrapartum stillbirth group (39.4%) than in the livebirth group (30.2%) experienced ruptured membrane on admission where the difference was statistically significant (p < 0.001).

Although over 84% of women in both intrapartum stillbirth and livebirth groups had relatively normal FHR (110-160) on admission, a significantly higher proportion of women in the intrapartum stillbirth group experienced FRH lower than 110/min. The result suggests foetal distress on admission. Accordingly, 13% of women in the intrapartum stillbirth group had foetal heart rate lower than 110/min on admission against only 0.8% of women in the livebirth (p < 0.001).

Data from the study population showed that over 97% of women in both intrapartum stillbirth and livebirth categories were examined for their cervical dilatation status on admission for the childbirth in review. Most women in both stillbirth and livebirth groups (over 61%) had cervical dilatation 4cm and above whereas 34.4% women in the stillbirth group and 38.7% in the livebirth group had cervical dilatation of 3cm and below on admissions to labour.

Proportionally, more women in the intrapartum stillbirth group (14.5%) than in the livebirth group (4.5%) had breech foetal presentation on admission to labour, the differences being statistically significant. The findings further revealed that data on foetal presentation during admission to labour was missing for approximately 13% of women in both intrapartum stillbirth and livebirth groups.

Table 2: Distribution of Labour Admission Assessment Indicators across Cases and Controls

| **Characteristics on admission** | **Stillbirth**  **N (%)** | **Live birth**  **N (%)** |  |
| --- | --- | --- | --- |
| **Status of Membrane** |  |  |  |
| Intact | 331 (46.8) | 895 (59.9) |  |
| Ruptured | 279 (39.4) | 452 (30.2) |  |
| Don't Know | 118 (13.8) | 204 (9.8) |  |
| **Foetal Heart Rate** |  |  |  |
| <110 | 97 (13.2) | 13 (0.8) |  |
| 110-160 | 632 (84.8) | 1524 (98.2) |  |
| >160 | 15 (2.0) | 14 (0.9) |  |
| **Status of Cervical Dilatation** |  |  |  |
| ≥3 | 250 (34.3) | 599 (38.7) |  |
| 4 | 108 (14.7) | 363 (23.23) |  |
| 5 | 85 (11.6) | 158 (10.2) |  |
| 6 | 69 (9.5) | 147 (9.5) |  |
| 7-10 | 218 (29.9) | 283 (18.2) |  |
| **Foetal presentation** |  |  |  |
| Vertex | 523 (71.9) | 1245 (81.2) |  |
| Breech | 106 (14.5) | 69 (4.5) |  |
| Shoulder | 3 (0.4) | 1 (0.1) |  |
| Don't know | 96 (13.2) | 236 (14.2) |  |

Multivariate logistic regression analysis was conducted to see if the obstetric conditions observed during admission could predict intrapartum stillbirth. Accordingly, the status of the foetal membrane, foetal heart rate and dilatation of the cervix were considered during analysis. Proportionally, more women in the stillbirth group (39.4%) than in the livebirth group (30.2%) experienced ruptured membrane on admission where the difference was statistically significant (p < 0.001). Women who had ruptured membrane on admission to labour were almost twice more likely to experience intrapartum stillbirth (OR 1.7, 95% CI 1.37–2.03). Women whose status of foetal membrane was missing in their obstetric records were more likely to experience intrapartum stillbirth (OR 1.80, 95% CI 1.36–2.40).

Women who were admitted for labour management in the public health facilities of Addis Ababa with diagnosis of foetal heart rate lower than 110/bpm were almost seven times more likely to experience intrapartum stillbirth (OR 6.96, 95% CI 2.75–17.66). On the contrary, women who were admitted with FHR in the range of 110–160/bpm had protective association against intrapartum stillbirth (aOR 0.37, 95% CI 0.15–0.92).

Data from this study revealed that most women (over 65%) in the intrapartum stillbirth group were admitted for labour with cervical dilatation of 4 cm or above and yet this was a predictor of intrapartum stillbirth (OR 1.2, 95% CI 1.00–1.45).

Results of clinical assessment on foetal presentation on admission for labour from this study indicated that proportionally more women in the livebirth group (81.2%) than in the intrapartum stillbirth group (71%) had normal (vertex) presentation. On the contrary, proportionally more women in the intrapartum stillbirth group (14.5%) than in the livebirth group (4.5%) had breech foetal presentation on admission to labour the difference being statistically significant (aOR 3.26 95% CI 1.93-5.50).

Table 3: Multiple Logistic Regression Analysis to Detect Independent Effects of Key Variable

| **Independent variable** | | | **Birth outcome** | | | | **Crude OR**  **(95% CI)** | | **Adjusted OR**  **(95% CI)** | |
| --- | --- | --- | --- | --- | --- | --- | --- | --- | --- | --- |
|  |  |  | **Stillbirth**  **N (%)** | | **Live birth**  **N (%)** | |  |  |  |  |
| **Gravida** | | |  | |  | |  | |  | |
| Nulliparous | | | 358 (49.2) | | 578 (37.3) | | 1.1 (0.67-1.8) | |  | |
| Multiparous | | | 370 (50.8) | | 973 (62.7) | | 0.61 (0.51-0.71) | | 0.73 (0.55-0.93) | |
| **Children alive** | | | | |  | |  | |  | |
| Zero |  | | 451 (68.8) | | 790 (55.2) | | 1.78 (1.47-2.17)** | | 1.48 (1.12-1.95)** | |
| One or more |  | | 205 (31.2) | | 640 (44.8) | | 1 | | 1 | |

| **Status of membrane on admission** | |  | |  | |  | |  | |
| --- | --- | --- | --- | --- | --- | --- | --- | --- | --- |
| Intact | | 331 (46.8) | | 895 (59.9) | | 1 | | 1 | |
| Ruptured | | 279 (39.4) | | 452 (30.2) | | 1.67 (1.37-2.03)** | | 1.18 (0.91-1.53) | |
| Don’t know | | 98 (13.8) | | 147 (9.8) | | 1.80 (1.36-2.40)** | | 1.51 (1.03-2.19)* | |
| **Foetal heart rate on admission** | |  | |  | |  | |  | |
| <110 | | 97 (13.2) | | 13 (0.8) | | 6.96 (2.75-17.66)** | | 5.63 (1.70-18.64)* | |
| 110-160 | | 624 (84.8) | | 1512 (98.2) | | 0.38 (0.18-0.80)* | | 0.37 (0.15-0.92) | |
| >160 | | 15 (2.0) | | 14 (0.9) | | 1 | | 1 | |
| **Cervical dilatation on admission** | |  | |  | |  | |  | |
| ≤4cm | | 252 (34.4) | | 593 (38.7) | | 1 | | 1 | |
| >4cm | | 481 (65.6) | | 940 (61.3) | | 1.20 (1.00-1.45)** | | 0.96 (0.75-1.24) | |
| **Foetal presentation on admission **** | |  | |  | |  | |  | |
| Vertex | | 525 (71.9) | | 1245 (81.2) | | 1 | | 1 | |
| None-vertex | | 109 (14.9) | | 70 (4.6) | | 3.69 (2.69-5.07)** | | 3.26 (1.93-5.50)** | |
| Don’t know | | 96 (13.2) | | 218 (14.2) | | 1.04 (0.80-1.36) | | 1.28 (0.87-1.89) | |

### **Discussion**

We observed that several variables including the status of cervical membrane, foetal heart rate, cervical dilatation and foetal presentation during admission to labour were associated with intrapartum stillbirth in public health facilities of Addis Ababa. Evidence shows that correct diagnosis of labour during admission to health facility for childbirth is critical to avoid any adverse outcomes or complications during delivery and immediate postnatal period. Several indicators including contraction of the uterus, cervical dilatation, status of membrane, and cervical effacement are among critical components for the definition of labour onset (5).

Our findings showed that women who were admitted with Premature Rapture of Membrane (PROM) had greater vulnerability to intrapartum stillbirth. PROM might be owing to delays in seeking obstetric care in the public health facilities because of socio-economic factors or ineffective inter-facility referral linkages. More alarming was the fact that data on the status of membrane was missing for relatively large proportion of cases which can be indicative of poor quality of obstetric service in correctly diagnosing and recording important indicators and procedures. The proportion of women with PROM on admission was higher compared to a study from India(9). It should be noted that premature rupture of membranes (PROM) at term (> 37 weeks) negatively affects between 8 and 10% of all pregnancies and misdiagnosing it at admission for labour could entail adverse outcomes including intrapartum stillbirth, pregnancy-related complications and maternal and foetal infection (4). Empirical evidence shows that PROM combined with subclinical chorioamnionitis was associated with foetal distress and stillbirth (10).

Evidence shows that lower FHR on admission results in sustained foetal distress during labour which increases the risk of intrapartum stillbirth (11). One of the most striking findings from this study was that cases with low foetal heart rate on admission were seven times more vulnerable to intrapartum stillbirth. FHR of lower than 110/bpm suggests the presence of foetal distress that warrants emergency obstetrical care. Treating these cases as obstetric emergency and providing responsive obstetric care services could have saved lives in this regard.

Measuring cervical dilatation is another routine intervention that helps determine admission decisions and can predict labour outcome. Women admitted with 3cm and less dilatation were comparable with a study based on the Danish dystocia research(12). Although Clotrida, et al. (2014:1) report concerns related to admission at a latent stage of labour, delays in seeking admission for labour could result in labour abnormalities potentially leading to adverse outcomes (7, 13). There has been a clinical consensus that the active phase of labour begins at approximately 4 cm cervical dilatation which is also a relatively good timing of admission for skilled delivery in the health facilities (14). Our findings showed that most women in the intrapartum stillbirth group were admitted for labour with cervical dilatation of 4 cm or above and yet this was a predictor of intrapartum stillbirth (OR 1.2, 95% CI 1.00–1.45) which might be owing to delayed health seeking by women who experienced intrapartum stillbirth.

Presentation refers to that part of the foetus entering the pelvic inlet first. The main presentations include shoulder, breech and cephalic (15). Cephalic presentation is the most physiologic and frequent foetal presentation and is associated with the highest rate of successful vaginal delivery as well as with the lowest frequency of complications (16).

Our findings related to foetal presentation on admission for labour showed that proportionally more women in the livebirth group than in the intrapartum stillbirth group had vertex whereas intrapartum stillbirth was three times higher among cases of non-vertex presentation. Foetal presentation is generally assessed by palpating the abdomen as part of a clinical examination, although its accuracy might vary depending on the provider and maternal factors. Correct detection of foetal presentation upon admission to labour could still reduce the risk of intrapartum stillbirth as immediate decisions to conduct caesarean section or to make emergency obstetric referral (17).

Our findings showed that considerable amount data was missing diagnosis of foetal presentation during admission to labour, which flags concern in relation to the quality of maternity care in the public health facilities of Addis Ababa. However, the findings were not specific enough as whether the missing data were owing to misdiagnosis or gaps in record keeping. However, the rates are very high compared to findings from a study in India (18).

The quality of data from the medical records in the public health facilities had certain gaps in terms of completeness, which can be referred as one of the limitations of this study. However, exclusion criteria were applied strictly to remove charts with incomplete records on the subject to reduce effects of these limitations.

**Conclusion**

Data on a few labour admission indicators including the status of membrane, FHR, cervical dilatation, and foetal presentation revealed statistically significant associations with intrapartum stillbirth. Accordingly, low FHR, non-vertex foetal presentations and ruptured cervical membrane were predictors of intrapartum stillbirth among the study population. Misdiagnosis and poor recording of labour admission assessment results were observed with association to intrapartum stillbirth. Health facilities could avert unnecessary foetal loss by undertaking timely and accurate diagnosis of key obstetric indicators during admission for labour in the public health facilities. More importantly, any unfavourable observations in this regard should be treated with outmost sensitivity including immediate referrals, labour induction, assisted delivery, or caesarean section depending on the appropriate clinical protocol.

**DECLARATIONS**

**Abbreviation**

AARHB: Addis Ababa Regional Health Bureau; ANC: Antenatal Care; ART: Ant-Retroviral Therapy; BEmONC: Basic Emergency Obstetric and Neonatal Care; CEmONC: Comprehensive Emergency Obstetric and Neonatal Care; HIV: Human Immuno-Deficiency Virus; WHO: World health Organization

**Ethical Approval**

Ethical approval was obtained from the Higher Degrees Committee of the University of South Africa (HSHDC/421/2015), and study permit was secured from health ethics committee of Addis Ababa Regional Health Bureau (AARHB) prior to data collection. The AARHB ethics committee approval and authorization letter from sub-city health offices granted permission to access patient charts without individual consents. Furthermore, individual identifiers including names and addresses of patients were excluded from data collection form to ensure case anonymity throughout the process.

**Consent to Publish**

Not Applicable

**Availability of Data and Materials**

Data and materials can be accessed through corresponding author.

**Competing Interests**

The authors declare that they had no competing interest on this study

**Funding**

The University of South Africa provided partial funding whereas the corresponding author covered the remaining expenses.

**Authors’ Contributions**

Both authors equally contributed to this study. As part of his PhD thesis, AG led the design, data collection, analysis, and drafting of the report for this study. As academic advisor, LM Modiba provided ongoing supervision, guidance and reviewed the study report as well as this manuscript.

**Acknowledgment**

University of South Africa partially funded the PhD research work without which this article would not have been produced.

**Authors’ details**

^1^ Chief of Party for an Integrated Nutrition and WASH Program, CRS, Rwanda, Kigali. Tel. +250788309289; e-mail- [alemayehug2000@gmail.com](mailto:alemayehug2000@gmail.com)

^2^ Department of Health Studies, University of South Africa, TvW 7-160 College of Human Sciences, Tel: 012 429 6337;E-mail: [modiblm@unisa.ac.za](mailto:modiblm@unisa.ac.za)

References

1. Bhutta ZA, Das JK, Bahl R, Lawn JE, Salam RA, Paul VK, et al. Can available interventions end preventable deaths in mothers, newborn babies, and stillbirths, and at what cost? *The Lancet,* 2014;384(9940):347-70.

2. Joy EL, Michael GG, Toni MN, Craig ER, Cynthia S. Global report on preterm birth and stillbirth: definitions, description of the burden and opportunities to improve data. *BMC Pregnancy and Childbirth,* 2010;10(Supp 1):1471-2393.

3. Lawn JE, Blencowe H, Pattinson R, Cousens S, Kumar R, Ibiebele I, et al. Stillbirths: Where? When? Why? How to make the data count? *The Lancet*, 2011;377(9775):1448-63.

4. Marshall J, Raynor M, editors. *Myles Textbook for Midwives*. Sixth ed. London: ELSEVIER; 2014.

5. Hanley GE, Munro S, Greyson D, Gross MM, Hundley V, Spiby H, et al. Diagnosing onset of labor: a systematic review of definitions in the research literature. *BMC Pregnancy Childbirth*, 2016;16(71):1-8.

6. Mikolajczyk RT, Zhang J, Grewal J, Chan LC, Petersen A, Gross MM. Early versus Late Admission to Labor Affects Labor Progression and Risk of Cesarean Section in Nulliparous Women. *Front Med* (Lausanne). 2016;3(26):1-5.

7. Clotrida C, Albert K, Dismas M, Marietha M. Labour management and Obstetric outcomes among pregnant women admitted in latent phase compared to active phase of labour at Bugando Medical Centre in Tanzania

*BMC Pregnancy & Childbirth,* 2014;14(68):1-5.

8. FMOH. *Health and Health related indicators*. Addis Ababa: FMOH; 2010.

9. Rahman H, Renjhen P, Dutta S, Kar S. Admission cardiotocography: Its role in predicting foetal outcome in high-risk obstetric patients. *Australas Med J.* 2012;5(10):522-7.

10. Zhang H, Wang LU, Wang J, Hei J, Ruan C. Premature rupture of the fetal membrane combined with subclinical chorioamnionitis negatively affects pregnancy outcomes by a mechanism associated with reduced levels of matrix metalloproteinase-2. *Exp Ther Med*. 2015;10(2):561-6.

11. Sandhu GS, Raju R, Bhattacharyya TK, Shaktivardhan. Admission Cardiotocography Screening of High Risk Obstetric Patients. *Medical Journal Armed Forces India*. 2008;64(1):43-5.

12. Kjaergaard H, Olsen J, Ottesen B, Nyberg P, Dykes AK. Obstetric risk indicators for labour dystocia in nulliparous women: a multi-centre cohort study. *BMC Pregnancy Childbirth,* 2008;8(45):1-6.

13. Wayu A, Yifru B. Predictors of labor abnormalities in university hospital: unmatched case control study. B*MC Pregnancy & Childbirth*, 2014;14(256):1-11.

14. Neal JL, Lowe NK, Ahijevych KL, Patrick TE, Cabbage LA, Corwin EJ. "Active labor" duration and dilation rates among low-risk, nulliparous women with spontaneous labor onset: a systematic review. *J Midwifery Womens Health*, 2010;55(4):308-18.

15. Kennedy B, McMurtry SB. *Intrapartum Management Modules*. Fifth Edition ed. Philadelphia: Wolters Kluwer; 2017. 822-3622 p.

16. Ferreira J, Borowski D, Czuba B, Cnota W, Wloch A, Sodowski K, et al. The evolution of fetal presentation during pregnancy: a retrospective, descriptive cross-sectional study. A*cta Obstet Gynecol Scand*, 2015;94(6):660-3.

17. Natasha N, Christine LR, Carolyn AC, Emily CO. Diagnostic accuracy of clinical examination for detection of non-cephalic presentation in late pregnancy: cross sectional analytic study. *BMJ,* 2006;333(7568):578-80.

18. Sharma G, Powell-Jackson T, Haldar K, Bradley J, Filippi V. Quality of routine essential care during childbirth: clinical observations of uncomplicated births in Uttar Pradesh, India. *Bulletin of the World Health Organization,* 2017;95(247):419-29.
